# Abnormalities of cerebral blood flow and the regional brain function in Parkinson’s disease: a systematic review and multimodal neuroimaging meta-analysis

**DOI:** 10.3389/fneur.2023.1289934

**Published:** 2023-12-07

**Authors:** Hao Xie, Yang Yang, Qian Sun, Ze-Yang Li, Min-Hua Ni, Zhu-Hong Chen, Si-Ning Li, Pan Dai, Yan-Yan Cui, Xin-Yu Cao, Nan Jiang, Li-Juan Du, Ying Yu, Lin-Feng Yan, Guang-Bin Cui

**Affiliations:** ^1^Department of Radiology and Functional and Molecular Imaging Key Lab of Shaanxi Province, Tangdu Hospital, Fourth Military Medical University (Air Force Medical University), Xi’an, Shaanxi, China; ^2^Faculty of Medical Technology, Xi’an Medical University, Xi’an, Shaanxi, China; ^3^Faculty of Medical Technology, Shaanxi University of Chinese Medicine, Xianyang, Shaanxi, China; ^4^Faculty of Medical Technology, Medical School of Yan’an University, Yan’an, Shaanxi, China

**Keywords:** functional neuroimaging, Parkinson’s disease, functional magnetic resonance imaging, cognition, coordinate-based meta-analysis

## Abstract

**Background:**

Parkinson’s disease (PD) is a neurodegenerative disease with high incidence rate. Resting state functional magnetic resonance imaging (rs-fMRI), as a widely used method for studying neurodegenerative diseases, has not yet been combined with two important indicators, amplitude low-frequency fluctuation (ALFF) and cerebral blood flow (CBF), for standardized analysis of PD.

**Methods:**

In this study, we used seed-based d-mapping and permutation of subject images (SDM-PSI) software to investigate the changes in ALFF and CBF of PD patients. After obtaining the regions of PD with changes in ALFF or CBF, we conducted a multimodal analysis to identify brain regions where ALFF and CBF changed together or could not synchronize.

**Results:**

The final study included 31 eligible trials with 37 data sets. The main analysis results showed that the ALFF of the left striatum and left anterior thalamic projection decreased in PD patients, while the CBF of the right superior frontal gyrus decreased. However, the results of multimodal analysis suggested that there were no statistically significant brain regions. In addition, the decrease of ALFF in the left striatum and the decrease of CBF in the right superior frontal gyrus was correlated with the decrease in clinical cognitive scores.

**Conclusion:**

PD patients had a series of spontaneous brain activity abnormalities, mainly involving brain regions related to the striatum-thalamic-cortex circuit, and related to the clinical manifestations of PD. Among them, the left striatum and right superior frontal gyrus are more closely related to cognition.

**Systematic review registration:**

https://www.crd.york.ac.uk/ PROSPERO (CRD42023390914).

## 1 Introduction

Parkinson’s disease (PD) is a neurodegenerative disease with the pathological characteristics of loss of dopamine neurons and aggregation of Lewy bodies (1). In the United States, Parkinson’s disease affects almost six per 1,000 people age 45 and over (2), and the incidence rate of Parkinson’s disease is increasing in most countries in the world (3, 4). Due to the increase in disease awareness, aging population, and environmental changes, the number of PD patients continues to increase, which has become a public health issue in aging societies across countries (5). The clinical manifestations of PD are divided into motor symptoms and non-motor symptoms. Motor symptoms include common symptoms such as tremor, bradykinesia, rigidity and gait disorders. With the deepening of research in recent years, cognitive disorders, sleep disorders, mood changes and other non-motor symptoms have gradually become the focus (6, 7). Especially cognitive impairment, which can lead to dementia, seriously affect patients’ health and increase social burden (8). Therefore, a better understanding of the neural substrates of cognitive impairment in PD is urgently required to direct effective and targeted treatment strategies.

In previous studies, researchers have tried many methods to clarify the physiological basis of PD, such as animal experiments, single photon emission computed tomography (SPECT) and resting state fMRI functional magnetic resonance imaging (rs-fMRI) (9, 10). Among them, rs-fMRI has been widely used in PD and other degenerative disease due to its advantages of non-invasive, efficient, and high spatial resolution imaging mode that can reflect the activities of the central nervous system. Amplitude of low-frequency fluctuations (ALFF) is an important indicator of rs-fMRI, obtained by measuring blood oxygen level dependent (BOLD) signals (11). Cerebral blood flow (CBF) is another important indicator obtained by measuring magnetic labeled endogenous arterial blood as a tracer using arterial spin labeling (ASL) technology (12, 13). The individual ALFF and CBF represent the neural activity and blood flow perfusion of the local brain region, respectively, and both indicators also reflect the intensity of neural activity in the brain region through direct and indirect means (14, 15). In addition, combining two indicators of the same brain region can reflect the neurovascular coupling state of a certain region (16, 17). Therefore, it is crucial to conduct research and analysis on these two indicators. Previous researchers have conducted years of research on PD based on ALFF and CBF, and have published many research results. However, due to differences in sample size, demographic information, ethnic distribution (many studies on East Asian populations), and clinical data, there may be significant heterogeneity and bias among studies. Some researchers have attempted to integrate the results using meta-analysis and review. For example, previous ALFF meta-analysis results have shown that PD patients have a decrease in ALFF in areas such as the left superior temporal gyrus and left superior frontal gyrus, while an increase in ALFF in areas such as the right superior frontal gyrus and left superior parietal lobule (18). The retrospective analysis of brain perfusion in PD patients using the ASL technique in the past suggests that the main brain regions related to motor and non-motor symptoms of PD, such as the basal ganglia subregion, frontoparietal network, and visual network, have been identified as insufficient CBF perfusion (19). The above results have significant differences and high heterogeneity due to differences in inclusion criteria and specific analysis methods in the literature. Therefore, it is essential to explore the brain regions affected by ALFF and CBF in PD using consistent methods and a more comprehensive analysis process. Based on this, we conducted this study.

The purpose of this study is to conduct multimodal meta-analysis of the changes of ALFF and CBF in PD compared with normal controls through whole-brain meta-analysis technology, and explore areas of the brain where ALFF, CBF, or both have changed, providing neuroimaging evidence for the clinical manifestations of PD, and attempting to identify neuroimaging biomarkers that lead to cognitive impairment, in order to assist in the early diagnosis and intervention of such patients.

## 2 Methods

### 2.1 Search strategy

The study followed the guidelines of the Preferred Reporting Items for Systematic Reviews and Meta-Analyses (PRISMA) and 10 simple rules for neuroimaging meta-analysis (20, 21). The protocol was registered in PROSPERO (CRD42023390914).^1^ Two databases were searched including PubMed, Web of Science, from Jan 1, 2007 to Dec 1, 2022. Based on the two indicators of ALFF and CBF, this search was divided into two parts. The first part used keywords: (“Parkinson’s Disease” OR “Parkinson Disease” OR “Parkinsonism” OR “Paralysis Agitans” OR “PD”) AND (“amplitude of low frequency fluctuation” OR “ALFF” OR “low frequency fluctuation” OR “LFF” OR “amplitude of low frequency oscillation” OR “LFO”). The second part used keywords: (“Parkinson’s Disease” OR “Parkinson Disease” OR “Parkinsonism” OR “Paralysis Agitans” OR “PD”) AND (“Cerebrovascular Circulation” OR “arterial spin labeling” OR “ASL” OR “Cerebral Blood Flow” OR “CBF”).

### 2.2 Study selection

After searching for studies, we first excluded duplicate studies. When reading and extracting information from the entire text, if there was any information not mentioned in the original text, such as coordinate values, non-online manuscripts, etc., the corresponding author would be contacted by email. Extracting information and conducting research, studies conforming to the following criteria were included: (1) the exploration of ALFF or CBF alterations between PD patients and healthy controls (HCs); (2) the subjects are adults; (3) PD patients were in an off-state; (4) the article clearly depicted the peak coordinates (Talairach or MNI) in the three-dimensional stereo directional coordinates; (4) available t values, *p* value or z values are provided in the study; (5) original research published in peer-reviewed journals. Studies conforming to the following criteria were excluded: (1) no HCs; (2) subjects with other diseases of central system or affecting brain activity; (3) animal study; (4) not related to ALFF and CBF; (5) studies with ROI analysis (6) research on minors; (7) secondary study; (8) neuroimaging quality score<16 or JBI score<12.

### 2.3 Quality assessment

We formulated the quality assessment method of this study referring to the previous high-quality research (22). Based on this, we used the checklist for objective evaluation of the quality of the neuroimaging meta-analysis research method (Supplementary Figure S1). In addition, the Joanna Briggs Institute (JBI) critical appraisal checklist (Supplementary Table S4) by the cross-sectional study was also used to conduct a secondary assessment of the quality of the included studies (23). Two reviewers (H.X and ZY.L, Radiologist) independently evaluated the quality of the article. If any differences were encountered during the process, the third reviewer (LF.Y, Deputy Chief Radiologist and Associate Professor) would make the final decision.

### 2.4 Voxel-wise meta-analysis of CBF and ALFF abnormalities

In this study, we used the SDM-PSI software (version 6.21^2^) to analyze studies on ALFF and CBF separately. SDM-PSI is a voxel based meta-analysis software that recreates brain maps comparing the effectiveness of results by using peak coordinates reported in research results and statistical effects extracted from each original study. The specific analysis process was reported in detail in previous articles (23–26). The main processes were briefly summarized: (1) global analysis; (2) pre-processing; (3) mean analysis; (4) threshold analysis; (5) family-wise error (FWE) correction; (6) threshold analysis and (7) extract peak coordinates and bias test. Finally, we used MICRON^3^ software to visualize the data results. The parameters used in this analysis were *p* < 0.005 uncorrected, minimum cluster extension>10 voxels and SDM-*Z* > 1 (which can reduce the possibility of false positive) (25). These parameters were recommended by the software developer and can best balance false positive and false results to obtain the best results (21, 25).

### 2.5 Heterogeneity, sensitivity and publication bias

In this study, we used Stata software to evaluate the heterogeneity of the results and conducted statistical analysis by extracting the peak coordinates of meaningful results. The results of the I^2^ statistic were used to evaluate the heterogeneity between studies. I^2^<50% usually indicates a low heterogeneity of the results (27). In order to determine whether there was potential publication bias, we conducted Egger’s test and drew a funnel map for visual inspection. Asymmetric funnel map or Egger’s test result *p* < 0.05 indicated that there was significant publication bias (28). The sensitivity analysis based on whole-brain voxel used AES-SDM software to test the reliability of the results by eliminating one data set at a time and then performing the same analysis method (22, 29). If a region was significant in most data set combinations (>50%), the result was considered highly reproducible and trustworthy.

### 2.6 Multimodal analysis of ALFF and CBF

Areas of shared abnormalities between patient groups versus control subjects of ALFF and CBF were determined in conjunction analyses by computing *p* value overlap within each voxel from the original meta-analytic maps accounting for error. Conjunction analysis determined overlapping (or distinct) regions between patient groups across both modalities.

### 2.7 Meta-regression analysis

Meta-regressions were conducted within the PD group to examine effects of age, course of disease, and clinical scale results on ALFF and CBF abnormalities. In order to minimize the false correlation, we adopted a low probability threshold of 0.0005. We ignored the results that do not exist in the main meta-analysis (30, 31).

## 3 Results

### 3.1 Included studies

**Figure 1:**
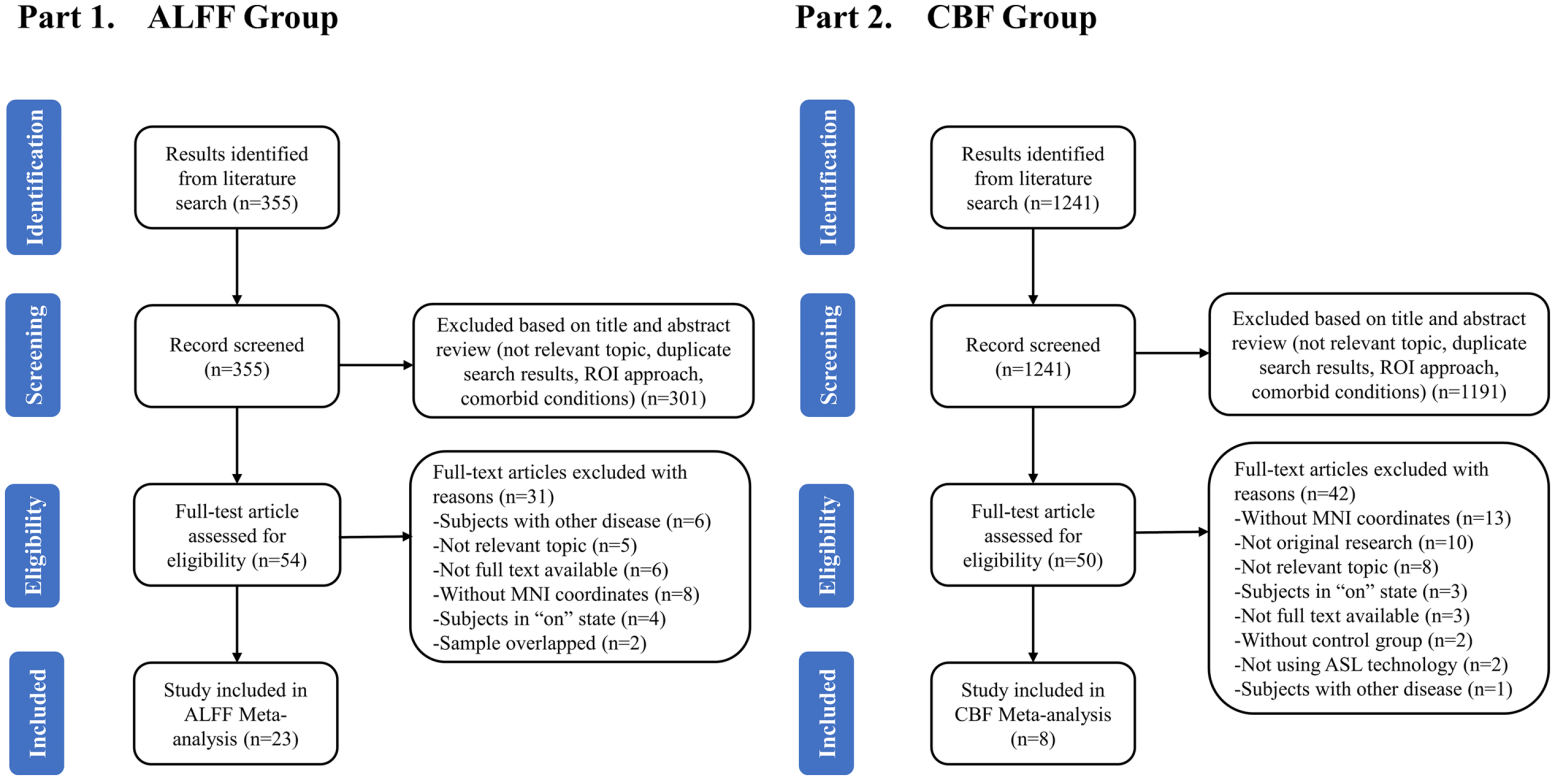
Flow diagram for identifying studies to be included in the meta-analysis.

Figure 1 showed the flowchart of this meta-analysis. After preliminary screening of titles and abstracts, a total of 104 articles (54 ALFF-related and 50 CBF-related) met the requirements. After reading the full text, 37 data sets of 31 studies were included, including 25 data sets for 23 ALFF-related studies and 12 data sets for 8 CBF-related studies. The quality score of the included study met the standard (Supplementary Tables S2, S3, S5).

### 3.2 Sample characteristics

Of the 23 original ALFF studies, 25 data sets reported the ALFF differences between 801 PD patients (457 males, 344 females, mean age = 60.3 years) and 738 healthy controls (382 males, 356 females, mean age = 59.8 years). Detailed demographic information, clinical features and imaging features were shown in Table 1 and Supplementary Table S6. After analyzing the extracted demographic and clinical information, we found that there were differences in gender distribution (*χ*2=4.338, *p* = 0.037), cognitive assessment scales MOCA (standardized mean difference [SMD] = −1.49; 95% confidence interval [CI] = [−1.91, −1.07], *Z* = 6.95, *P* < 0.00001), and MMSE (SMD = −0.49; 95% CI = [−0.66, −0.31], *Z* = 5.44, *P* < 0.00001) between the two groups. There was no difference between the two groups in terms of age (SMD = 0.57; 95% CI = [−0.18, 1.32], *Z* = 1.48, *p* = 0.14) and years of education (SMD = 0.33; 95% CI = [−0.28, 0.93], *Z* = 1.05, *p* = 0.29).

**Table 1:** Demographic and clinical characteristics of PD patients and HCs included in the meta-analysis of studies on ALFF.

Study	Indicator	Subjects (male/female)		Mean age (SD)		H-Y stage	UPDRS III score	Duration years (SD)	MMSE (SD)		Education years (SD)		MoCA (SD)	
		PD	HC	PD	HC				PD	HC	PD	HC	PD	HC
Harrington et al. (32)	ALFF	31(22/9)	30(11/19)	67.4(7.5)	68.6(7.2)	/	38.1(14.6)^a^	5.4(3.8)	29.3(0.9)	29.5(0.7)	17.0(2.3)	16.5(1.8)	/	/
Hou et al. (33)	ALFF	101(59/42)	102(60/42)	59.8(7.2)	59.9(7.1)	1.9(0.7)	25.5(11.5)^a^	7.2(4.4)	28.7(1.3)	29.0(1.1)	/	/	/	/
Kwak et al. (34)	ALFF	24(22/2)	24(19/5)	64.3(8.0)	63.3(7.0)	2.2(0.3)	18.5(8.0)^a^	5.4(3.0)	28.8(1.0)	29.1(1.0)	/	/	26.0(3.0)	25.7(3.0)
Li et al. (35)	ALFF	16(6/10)	19(11/8)	62.8(6.6)	62.7(8.1)	2.2(0.8)	22.1(12.5)	4.0(4.3)	27.6(2.4)	/	/	/	/	/
Luo et al. (36)	ALFF	37(17/20)	13(6/7)	61.5(9.5)	62.5(9.6)	/	38.2(14.9)	/	26.8(2.9)	28.1(1.8)	/	/	24.1(4.6)	28.8(1.1)
Luo et al. (37)	ALFF	30(15/15)	30(15/15)	53.6(10.2)	51.9(7.7)	1.7(0.6)	26.8(12.4)	2.1(1.3)	27.0(2.8)	28.1(1.8)	/	/	/	/
Mi et al. (38)	ALFF	31(20/11)	32(17/15)	58.0(9.8)	58.3(7.3)	1.9(0.6)	30.8(14.4)	5.2(3.5)	27.8(1.7)	28.6(1.4)	/	/	24.8(3.0)	26.2(3.4)
Rong et al. (39)	ALFF	42(26/16)	33(17/16)	64.5(6.7)	63.3(5.3)	2.0(0.5)	31.2(13.2)	2.9(2.2)	28.2(1.9)	28.6(1.5)	10.8(3.3)	11.4(3.2)	23.3(3.3)	26.2(1.9)
Skidmore et al. (40)	ALFF	14(11/3)	15(9/6)	62.0(9.0)	65.0(13.0)	/	37.0(13.0)	/	27.0(3.0)	28.0(3.0)	/	/	25.0(3.0)	27.0(3.0)
Sun et al. (41)	ALFF	26(14/12)	23(13/10)	59.6(10.0)	59.5(10.8)	1.9(0.6)	17.1(3.7)	2.0(0.9)	25.9(4.3)	25.6(4.4)	7.1(5.1)	7.9(6.0)	24.0(5.4)	23.6(6.3)
Tang et al. (42)	ALFF	51(27/24)	50(21/29)	53.2(11.0)	51.5(10.7)	2.4(0.8)	48.6(23.4)^a^	5.8(5.0)	27.1(3.6)	27.8(3.2)	/	/	22.4(5.7)	25.2(5.0)
Wang et al. (43)	ALFF	17(9/8)	25(14/11)	62.5(10.2)	64.7(5.2)	2.4(0.6)	48.4(13.9)	6.5(3.6)	24.8(4.2)	28.5(1.5)	10.8(4.2)	11.5(3.0)	19.7(5.8)	26.2(1.4)
Wang et al. (44)	ALFF	33(24/9)	19(10/9)	69.5(6.0)	66.2(3.5)	2.4(0.6)	22.7(10.8)	4.4(3.0)	28.1(1.7)	28.8(1.0)	11.5(4.0)	10.6(3.2)	/	/
Wang et al. (45)	ALFF	10(5/5)	13(11/2)	64.7(7.0)	62.9(9.0)	1.5(0.5)	29.3(9.6)^a^	5.4(7.9)	/	/	/	/	24.6(2.4)	27.9(1.1)
Wang et al. (45)	ALFF	19(14/5)	13(11/2)	59.1(12.3)	62.9(9.0)	1.3(0.5)	25.7(11.8)^a^	9.5(10.8)	/	/	/	/	28.4(1.3)	27.9(1.1)
Wen et al. (46)	ALFF	16(8/8)	21(13/8)	60.7(18.7)	55.4(16.4)	1.6(1.0)	33.8(24.2)	5.6(7.4)	29.2(2.2)	/	/	/	/	/
Xiang et al. (47)	ALFF	24(12/12)	22(11/11)	62.7(7.4)	65.6(6.9)	2.2(0.9)	22.0(7.0)	7.0(3.3)	27.3(2.1)	28.6(1.6)	13.6(3.1)	12.9(3.7)	25.9(3.7)	25.4(2.5)
Xu et al. (48)	ALFF	19(11/8)	32(13/19)	60.3(11.3)	63.2(4.7)	1.4(0.5)	26.9(13.5)	3.2(3.3)	27.1(1.9)	28.7(1.3)	11.2(4.8)	9.8(3.8)	/	/
Yao et al. (49)	ALFF	12(4/8)	14(6/8)	63.4(7.4)	64.1(4.0)	2.8(0.9)	18.0(12.9)	8.4(5.1)	28.5(1.7)	29.1(0.7)	/	/	/	/
Yue et al. (50)	ALFF	26(16/10)	10(3/7)	60.8(3.5)	58.0(4.1)	1.4(0.6)	33.7(14.0)	1.8(1.2)	25.9(3.6)	27.9(1.3)	/	/	22.0(4.2)	26.5(3.2)
Yue et al. (50)	ALFF	14(8/6)	10(5/5)	49.8(3.6)	49.7(2.3)	1.7(0.6)	34.7(10.6)	2.2(1.4)	26.4(4.4)	26.4(2.7)	/	/	24.4(4.4)	24.6(3.7)
Zhang et al. (51)	ALFF	28(15/13)	28(14/14)	59.2(9.7)	58.2(6.5)	2.0(0.7)	29.1(8.7)	8.5(2.9)	27.6(1.3)	27.7(1.2)	/	/	24.4(2.5)	25.9(1.7)
Zhang et al. (52)	ALFF	82(35/47)	77(31/46)	59.7(11.9)	58.6(8.5)	/	20.2(8.4)	7.1(6.0)	/	/	/	/	/	/
Zhang et al. (53)	ALFF	32(22/10)	25(12/13)	65(8.4)	64.6(4.5)	2.2(0.7)	21.6(10.0)	4.0(4.0)	28.3(1.8)	/	11.4(3.5)	10.7(2.9)	/	/
Zhang et al. (54)	ALFF	66(35/31)	58(29/29)	53.8(10.7)	51.7(10.8)	2.3(0.8)	30.7(15.3)	/	26.8(3.7)	27.9(3.3)	/	/	/	/

Data are presented as mean (SD). PD, Parkinson’s disease; HCs, healthy controls; SD, standard deviation; H-Y stage, Hoehn-Yahr stage; UPDRS III score, unified Parkinson’s disease rating scale III score, MMSE, mini-mental state examination; MoCA, montreal cognitive assessment.

^a^Only UPDRS total scores was given in the study.

/ means no relevant information was provided in the study.

A total of 12 data sets were obtained from the 8 studies on CBF, which included 285 PD patients (155 males, 130 females, mean age = 62.3 years) and 302 healthy controls (154 males, 148 females, mean age = 60.4 years). Detailed demographic information, clinical features and imaging features were shown in Table 2 and Supplementary Table S7. After analyzing the extracted demographic and clinical information, we found that there were differences in age distribution (SMD = -1.56; 95% CI = [0.38, 2.75], *Z* = 2.59, *p* = 0.01), cognitive assessment scales MOCA (SMD = -2.54; 95% CI = [−3.10, −1.98], *Z* = 8.85, *P*<0.00001), and MMSE (SMD = -2.48; 95% CI = [−2.81, −2.16], *Z* = 15.05, *P*<0.00001) between the two groups. There was no difference between the two groups in terms of gender (*χ*2 = 0.677, *p* = 0.411) and years of education (SMD = −0.46; 95% CI = [−0.98, 0.07], *Z* = 1.71, *p* = 0.09).

**Table 2:** Demographic and clinical characteristics of PD patients and HCs included in the meta-analysis of studies on CBF.

Study	Indicator	Subjects (male/female)		Mean age (SD)		H-Y stage	UPDRS III score	Duration years (SD)	MMSE (SD)		Education years (SD)		MoCA (SD)	
		PD	HC	PD	HC				PD	HC	PD	HC	PD	HC
Arslan et al. (55)	CBF	26(16/10)	15(11/4)	60.2(9.0)	58.7(6.3)	1.8(0.5)	26.0(10.8)	5.4(3.0)	29.4(0.8)	30.0(0.0)	10.1(3.8)	11.0(3.8)	26.7(1.9)	25.2(2.3)
Arslan et al. (55)	CBF	27(21/6)	15(11/4)	64.0(8.1)	58.7(6.3)	1.9(0.6)	32.4(13.1)	6.6(3.5)	28.2(1.4)	30.0(0.0)	9.0(3.8)	11.0(3.8)	22.4(2.5)	25.2(2.3)
Barzgari et al. (56)	CBF	30(24/6)	31(25/6)	66.1(9.0)	67.5(8.3)	1.8(0.7)	20.3(10.6)	6.1(3.9)	/	/	/	/	/	/
Jia et al. (57)	CBF	27(15/12)	25(11/14)	63.1(6.6)	59.4(5.8)	1.9(0.5)	23.8(7.4)	3.7(3.0)	/	/	13.4(2.9)	12.1(2.9)	/	/
Jia et al. (57)	CBF	27(16/11)	25(11/14)	62.6(6.6)	59.4(5.8)	1.7(0.7)	21.4(9.5)	3.7(2.9)	/	/	7.8(2.9)	12.1(2.9)	/	/
Lin et al. (58)	CBF	20(6/14)	22(7/15)	63.3(6.4)	59.9(6.0)	2.0(0.8)	22.9(15.1)	2.5(1.5)	22.6(7.4)	27.1(2.1)	8.8(4.9)	11.5(4.9)	/	/
Lin et al. (59)	CBF	17(6/11)	17(8/9)	63.7(8.5)	59.7(7.5)	2.0(2.0)	19^a^	3.5(2.4)	21.5(4.7)	28.7(2.0)	7.1(6.0)	10.2(6.0)	/	/
Lin et al. (59)	CBF	17(7/10)	17(8/9)	60.9(10.2)	59.7(7.5)	2.0(2.0)	17^a^	2.5(1.5)	28.3(2.3)	28.7(2.0)	9.3(6.0)	10.2(6.0)	/	/
Shang et al. (60)	CBF	42(18/24)	50(25/25)	66.8(8.4)	68.2(4.1)	1.8(0.9)	42.4(14.2)	2.2(1.0)	25.2(0.7)	28.3(1.2)	13.3(2.2)	13.2(2.2)	22.0(1.9)	28.3(1.4)
Suo et al. (61)	CBF	22(9/13)	36(15/21)	53.8(8.5)	53.7(7.3)	1.9(0.6)	23.2(10.2)	2.4(1.7)	27.6(2.1)	28.2(1.6)	10.3(2.9)	10.8(2.9)	19.1(2.9)	23.1(2.5)
Suo et al. (61)	CBF	17(10/7)	36(15/21)	54.0(8.2)	53.7(7.3)	1.8(0.6)	17.1(9.7)	2.2(1.6)	27.8(4.4)	28.2(1.6)	11.3(2.9)	10.8(2.9)	24.3(2.4)	23.1(2.5)
Zhao et al. (62)	CBF	13(7/6)	13(7/6)	61.9(9.2)	62.3(8.1)	1.4(0.5)	14.2(3.9)	3.8(2.9)	28.6(1.9)	28.8(1.1)	11.2(5.6)	13.0(5.6)	/	/

Data are presented as mean (SD). PD, Parkinson’s disease; HCs, healthy controls; SD, standard deviation; H-Y stage, Hoehn-Yahr stage; UPDRS III score, unified Parkinson’s disease rating scale III score; MMSE, mini-mental state examination; MoCA, montreal cognitive assessment.

^a^Only the mean value of the data was given in the study.

/ means no relevant information was provided in the study.

### 3.3 Meta-analysis of ALFF

**Figure 2:**
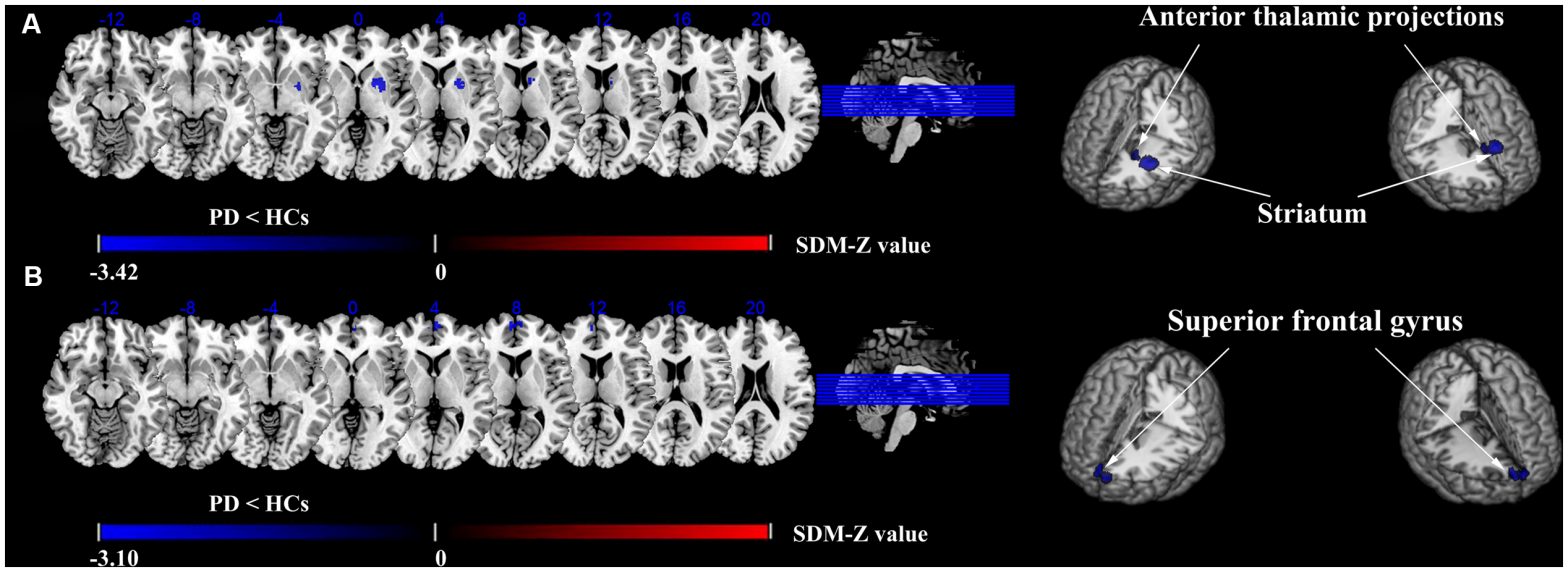
Differences in ALFF and CBF between PD and HCs groups. Meta-analyses results regarding. **(A)** ALFF difference between PD and HCs, **(B)** CBF difference between PD and HCs.

**Figure 3:**
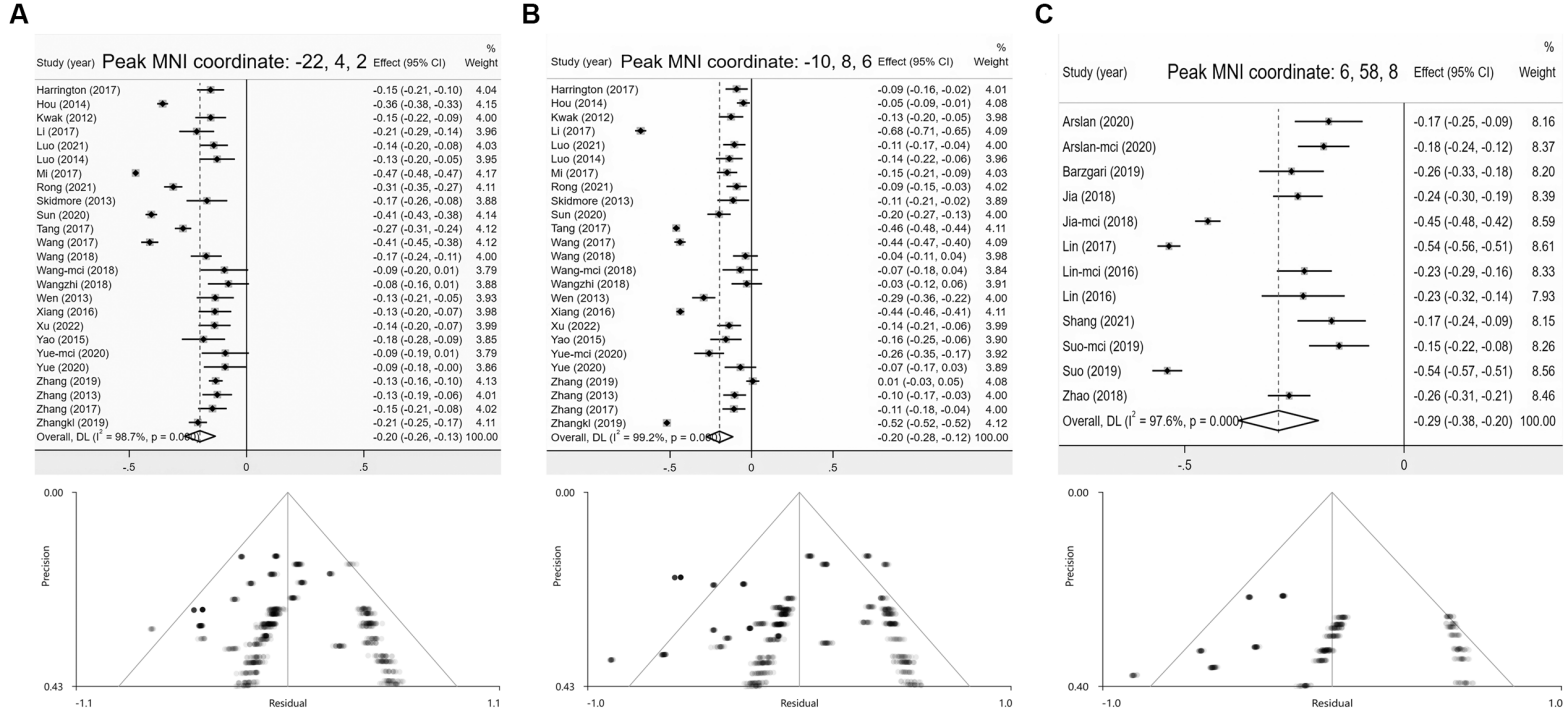
Forest and funnel plots of peak MNI coordinates. **(A,B)** Peak MNI coordinates of clusters with reduced ALFF in PD group; **(C)** Peak MNI coordinates of clusters with reduced CBF in PD.

Compared with HCs, the ALFF of PD patients decreased in the left striatum (BA 48, MNI: −22, 4, 2; SDM-Z = −3.420, *p* < 0.0005) and the left anterior thalamic projections (BA 25, MNI: −10, 8, 6; SDM-Z = −2.731, *p* < 0.005) (Figure 2; Table 3). Compared with HCs, there was no area of increased ALFF in PD patients. These regions showed significant between-study heterogeneity, but there was no publication bias. The jackknife analysis results also suggested that they can be reproduced in most combinations. The forest map for assessing heterogeneity and the funnel map for assessing publication bias were shown in Figure 3.

**Table 3:** Differences between PD patients and HCs.

Brain areas	MNI coordinate	SDM-Z	*p*-value	Voxels	Breakdown (voxels)	Egger’s test (*p* value)	Heterogeneity (*I*^2^)	Jackknife analysis
PD<HCs in ALFF								
Left striatum	−22, 4, 2	−3.420	<0.0005	168	Left striatum (112)	0.709	98.70%	25/25
					Left lenticular nucleus, putamen, BA 48 (32)			
					Left lenticular nucleus, putamen (22)			
					Left pons (2)			
Left anterior thalamic projections	−10, 8, 6	−2.731	<0.005	22	Left anterior thalamic projections (16)	0.951	99.20%	23/25
					Left caudate nucleus (4)			
					Left caudate nucleus, BA 25 (2)			
PD<HCs in CBF								
Right superior frontal gyrus, medial, BA 10	6, 58, 8	−3.102	<0.001	88	Left superior frontal gyrus, medial, BA 10 (39)	0.235	97.60%	11/12
					Right superior frontal gyrus, medial, BA 10 (28)			
					Left superior frontal gyrus, medial (9)			
					Left anterior cingulate, BA 10 (6)			
					Right superior frontal gyrus, medial (3)			

PD, Parkinson’s disease; HCs, healthy controls; ALFF, amplitude of low frequency fluctuation, CBF, cerebral blood flow.

### 3.4 Meta-analysis of CBF

Compared with HCs, the CBF index of PD patients decreased in the right superior frontal gyrus (R-SFG) (BA 10, MNI: 6, 58, 8; SDM-Z = −3.102, *p* < 0.001) (Figure 2; Table 3). Compared with HCs, there was no area of increased CBF in PD patients. The result of this brain area test showed that there was great heterogeneity, but there was no publication bias, and the jackknife analysis results also suggested that the results could be replicated in most combinations. The forest map for assessing heterogeneity and the funnel map for assessing publication bias were shown in Figure 3.

### 3.5 Multimodal analysis

PD patients relative to HCs showed no brain areas where ALFF and CBF increased or decreased simultaneously, nor did they showed any brain areas where ALFF increased and CBF decreased or ALFF decreased and CBF increased.

### 3.6 Meta-regression analysis

In the meta-analysis, the effects of potential risk factors (e.g., age, female proportion, duration of disease, MOCA score, MMSE score, H-Y stage and UPDRS III score) on the main results of the ALFF and CBF meta-analysis were studied, respectively. Regression analysis showed that the age, female proportion, course of disease, H-Y stage and UPDRS III score of PD patients had no effect on the main results, while lower MOCA score and MMSE score were associated with lower ALFF at the left striatum (Peak MNI coordinate: −18, 4, −2) and lower CBF at the R-SFG (Peak MNI coordinate: 6, 58, 8).

## 4 Discussion

In this study, we combined the brain nerve activity and cerebral blood flow changes of PD for the first time, and the correction method that best balances false positive and false negative results was selected for this systematic review and meta-analysis. The main results showed that the ALFF of PD in the left striatum and left anterior thalamic projections decreased, representing a decrease in neural activity. In the R-SFG, the CBF of PD decreased, which represents the decrease of local cerebral perfusion. In addition, the results of meta-analysis showed that the decrease in clinical cognitive scale scores was related to the decrease of ALFF in the left striatum and the decrease of CBF in the R-SFG. Jackknife analysis showed that the peak coordinates of the above results had high repeatability proving that the results were stable.

It is well known that the previous pathophysiology interpretation of PD focused on the degeneration of dopaminergic substantia nigra striatum neurons, because the striatum, as an important input node in the basal ganglia, played an important role in the control and output of movement (63–66). In particular, the changes in the dorsal striatum (caudate-putamen), because it received information directly from the dopaminergic neurons in the substantia nigra, the impairment of the activity of the dorsal striatum can lead to the dysfunction of the striatum-thalamus-cortex circuit (67–70), leading to a series of clinical manifestations of motor symptoms (71–73). In addition, a large number of studies observed that in addition to motor symptoms, PD patients also suffered from cognitive disorders, such as executive ability, working memory, planning strategies and attention set switching disorders (74–77). Further research has found that the cognitive impairment of PD patients was also closely related to the functional damage of striatum dopaminergic neural pathway. Previous pathological and PET studies have found that the dysfunction of the dopamine system in the caudate-putamen of the striatum was related to some features of cognitive impairment (78–80). And some structural MRI studies confirmed that compared with HCs, the caudate-putamen volumes of patients with PD accompanied by cognitive impairment were reduced (81, 82). The above results suggest that the striatum not only plays an important role in motor symptoms through the striatum-thalamus-cortex circuit in PD patients, but also may play an important role in cognitive impairment.

The results of this meta-analysis showed that the ALFF in the left striatum and left anterior thalamus projection in PD patients was significantly reduced. After the subdivision of the region, the left putamen and left caudate were included, and the putamen mass voxels were the largest. The above brain regions are important nodes in the striatum-thalamus-cortical circuit. The changes in ALFF have also been confirmed in previous studies to be related to motor disorders in PD (64, 73), which is also consistent with the physiological basis of the clinical motor symptoms of PD originating from the changes of dopaminergic neurons in the substantia nigra and striatum (68, 69). Other than motor symptoms, the neural activity of striatum has also been confirmed to be related to cognitive ability and learning ability by various experimental methods (83, 84). The results of this regression analysis showed that the change of ALFF in the left striatum was related to the change of cognitive score, which also suggested that striatum neuronal activity participated in the modulation of cognitive function in PD patients. In particular, putamen, in previous fMRI studies of PD, other studies also showed that putamen CBF decreased, and voxel-based morphological measurements showed volume reduction (82, 85). Previous studies have confirmed that the reliable reduction of ALFF in putamen was related to the increase of PD severity, and even suggested that the change of putamen can be defined as the imaging evidence of PD (42, 86, 87).

The results of the meta-analysis of CBF studies suggested that the CBF of PD in the R-SFG was lower than that of healthy controls. The R-SFG is also a part of the striatum-thalamus-cortex circuit, and its role was more related to cognition (73, 88). It is generally believed that this region is related to high-level cognitive functions, such as inductive reasoning, computation, and also responsible for working memory and procedural learning (89–91). The decrease in CBF in this region may be related to a significant statistical difference in cognitive scores between the two groups on baseline information. Regression analysis results showed that the reduced CBF in R-SFG was correlated with lower cognitive scores in the PD group on baseline information, which confirmed this hypothesis.

Combined with the regression analysis results of ALFF and CBF, the left striatum and R-SFG in the main results would be affected after the cognitive scale scores in the baseline information were added to the regression, which indicates that the above brain regions participate in the feedback of cognitive activities to the center in PD. The Striatum and prefrontal cortex are important components of dopamine’s mesocortical pathway, which is one of the three parallel pathways of dopamine, and is responsible for executive functions closely related to cognitive ability (92). Impairment of pathways can affect patients’ cognitive function. Another study in intraoperative stimulation and diffusion tomography provides direct evidence for the involvement of fiber bundle pathways in striatum and prefrontal cortex in cognitive control (93).

The results of multimodal analysis showed that PD patients did not have brain regions where ALFF and CBF increased and decreased at the same time or one of them increased and the other decreased. It was previously believed that ALFF and CBF were independent indicators, but some studies have shown that ALFF calculated from BOLD signals can be regulated by changes in CBF (94). Due to the fact that only a single changed in ALFF or CBF resulted in coupling changes in ALFF-CBF in the left striatum, left anterior thalamic projections and R-SFG, we believed that neurovascular uncoupling occurred in the main outcome.

Neurovascular coupling describes the close temporal and regional connection between cerebral blood flow response and neural activity, and the consistency of coupling can quickly provide sufficient nutrition and eliminate metabolic waste (95, 96). Research had confirmed that the state of neurovascular coupling changes with age and was related to executive function (97). Combining fMRI indicators to evaluate neurovascular coupling has been widely used in clinical research, especially in the fields of cognitive impairment and dementia (17, 98). For PD patients, abnormal neuronal activity caused by dopamine or other non-dopamine dysfunction, as well as perfusion damage caused by blood–brain barrier disruption, can cause changes in neurovascular coupling during the progression of the disease (99–101). The occurrence of this state may lead to toxic molecules entering the brain due to changes in vascular permeability, or obstacles in the clearance of local metabolites leading to neuronal dysfunction, thereby playing a role in neurodegenerative diseases or cognitive disorders (102). Some researchers analyzed the neurovascular decoupling state of PD with cognitive impairment (103), and the results showed that the uncoupling region included the left striatum and the right frontal lobe, and participated in the regulation of PD cognitive impairment, which was consistent with the results of this study. In addition, another study showed that neurovascular decoupling in the visual cortex of PD patients was associated with visual functional impairment, and it was confirmed that changes in neurovascular coupling state were not related to changes in gray matter volume (GMV) after regression (104). GMV had always been an important confounding factor in neuroimaging, and the above studies suggested that neurovascular coupling may be a potential analysis indicator unaffected by GMV, with broader application prospects.

Finally, there are some limitations in this meta-analysis. First, this study only included the literature that provided the peak coordinates, and excluded those that were not provided, which is also a common defect in the meta-analysis of neuroimaging studies (21, 26). Secondly, most of the research groups included are East Asian people, and the universality of the results is limited. In the future, it is necessary to enrich the database and update the meta-analysis to make the population more diverse, and the results have better applicability to different populations. Third, the analysis of concomitant cognitive impairment could not be sub-group analysis because there were few studies on clear diagnosis. Fourth, although we suspect that high heterogeneity may be caused by differences in GMV of subjects and software selection, parameter settings, and correction methods during data processing (Supplementary Tables S6–S7), there is not enough data to correct for these differences. Fifth, lack of data to further explore the potential neural mechanisms underlying the occurrence of various subtypes of cognition, such as memory, executive function, language, and abstract thinking, etc.

## 5 Conclusion

Compared with healthy controls, there was a series of brain areas with spontaneous abnormal brain activity in PD, mainly involving the striatum-thalamic-cortical circuit, which was related to the clinical symptoms related to movement disorders and cognitive decline. Specifically, the left striatum and left anterior thalamic projection ALFF decreased, and the right superior frontal gyrus CBF decreased. The left striatum and right superior frontal gyrus were more closely related to cognition. In conclusion, our study provides a reference for further exploring the changes of brain activity and the mechanism of cognitive impairment in PD.

## Data availability statement

The original contributions presented in the study are included in the article/Supplementary material, further inquiries can be directed to the corresponding authors.

## Author contributions

HX: Data curation, Formal analysis, Investigation, Methodology, Software, Visualization, Writing – original draft, Writing – review & editing. YaY: Investigation, Software, Supervision, Validation, Writing – original draft. QS: Data curation, Investigation, Visualization, Writing – original draft. Z-YL: Data curation, Software, Validation, Writing – original draft. M-HN: Investigation, Writing – original draft. Z-HC: Software, Writing – original draft. S-NL: Data curation, Writing – original draft. PD: Investigation, Writing – original draft. Y-YC: Visualization, Writing – original draft. X-YC: Data curation, Writing – original draft. NJ: Investigation, Writing – original draft. L-JD: Supervision, Writing – original draft. YiY: Supervision, Writing – original draft. L-FY: Conceptualization, Funding acquisition, Supervision, Validation, Writing – review & editing. G-BC: Conceptualization, Project administration, Resources, Supervision, Validation, Writing – review & editing.

## Footnotes

^1^ https://www.crd.york.ac.uk/prospero/

^2^ https://www.sdmproject.com/

^3^ https://www.nitrc.org/projects/bnv/
