# Supplementary material for: Abnormalities of cerebral blood flow and the regional brain function in Parkinson’s disease: a systematic review and multimodal neuroimaging meta-analysis
Source: Front Neurol. 2023 Dec 7;14:1289934. doi: 10.3389/fneur.2023.1289934 (PMC10755479; doi:10.3389/fneur.2023.1289934)
Supplement: Supplementary file 1 [file Data_Sheet_1.docx]

**Supplementary Materials**

| **Table S1. The detailed search strategy for the database** | | |
| --- | --- | --- |
| **Database** | **Search strategy** | |
|  | **Part 1** | **Part 2** |
| **PubMed** | ((((((Parkinson’s Disease[MeSH Terms]) OR (Parkinson’s Disease[Text Word])) OR (Parkinson Disease[Text Word])) OR (Parkinsonism[Text Word])) OR (Paralysis Agitans[Text Word])) OR (PD[Text Word])) AND ((((((amplitude of low frequency fluctuation[Text Word]) OR (ALFF[Text Word])) OR (low frequency fluctuation[Text Word])) OR (LFF[Text Word])) OR (amplitude of low frequency oscillation[Text Word])) OR (LFO[Text Word])) | ((((((Parkinson’s Disease[MeSH Terms]) OR (Parkinson’s Disease[Text Word])) OR (Parkinson Disease[Text Word])) OR (Parkinsonism[Text Word])) OR (Paralysis Agitans[Text Word])) OR (PD[Text Word])) AND (((((Cerebrovascular Circulation[MeSH Terms]) OR (arterial spin labeling[Text Word])) OR (ASL[Text Word])) OR (Cerebral Blood Flow[Text Word])) OR (CBF[Text Word])) |
| **Web of science** | (((((TS=(Parkinson’s Disease)) OR TS=(Parkinson Disease)) OR TS=(Parkinsonism)) OR TS=(Paralysis Agitans)) OR TS=(PD)) and ((((((TS=(amplitude of low frequency fluctuation)) OR TS=(ALFF)) OR TS=(low frequency fluctuation)) OR TS=(LFF)) OR TS=(amplitude of low frequency oscillation)) OR TS=(LFO)) | (((((TS=(Parkinson’s Disease)) OR TS=(Parkinson Disease)) OR TS=(Parkinsonism)) OR TS=(Paralysis Agitans)) OR TS=(PD)) and (((((TS=(Cerebrovascular Circulation)) OR TS=(arterial spin labeling)) OR TS=(ASL)) OR TS=(Cerebral Blood Flow)) OR TS=(CBF)) |

**Figure S1. Checklist for objective assessment of methodological quality of studies for neuroimaging meta-analysis**

| Category 1: Sample characteristics (10) |
| --- |
| 1. Patients were evaluated with specific standardised diagnostic criteria (1) |
| 2. Important demographic data (age and gender) were reported with mean (or median) and standard deviations (or range)) (2) |
| 3. Healthy comparison subjects were evaluated to exclude psychiatric and medical illnesses and demographic data was reported (1) |
| 4. Important clinical variables (eg. illness duration, onset time, medication status, HAMD scores) were reported with mean (or median) and standard deviations (or range)) (4) |
| 5. Sample size per group > 10 (2) |
| Category 2: Methodology and reporting (10) |
| 1. Whole brain analysis was automated with no a-priori regional selection (3) |
| 2. Magnet strength at least 1.5T (1) |
| 3. At least 5 minutes of resting state acquisition (1) |
| 4. Whole brain coverage of resting scans (1) |
| 5. The acquisition and preprocessing techniques were clearly described so that they could be reproduced (1) |
| 6. Coordinates reported in a standard space (1) |
| 7. Significant results are reported after correction for multiple testing using a standard statistical procedure (FDR, FWE or permutation-based methods) (1) |
| 8. Conclusions were consistent with the results obtained and the limitations were discussed (1) |

A maximum score of 20 for each study, allocated as per the criteria specified above.

**Table S2.** **Objective assessment of quality of included studies. (Group ALFF)**

|  | **Sample characteristics (10)** | | | | | | **Methodology and reporting (10)** | | | | | | | |  |
| --- | --- | --- | --- | --- | --- | --- | --- | --- | --- | --- | --- | --- | --- | --- | --- |
| **Study** | standardised diagnostic criteria | Important demographic data (age and gender) | | Healthy comparison subjects | Important clinical variables | Sample size per group > 10 | Whole brain analysis was automated | Magnet strength at least 1.5T | At least 5 minutes of resting state acquisition | Whole brain coverage of resting scans | The acquisition and preprocessing techniques were clearly described | Coordinates reported in a standard space | FDR, FWE or permutation-based methods | Conclusions were consistent with the results | Quality score |
| Harrington | **●** | **●●** | **●** | | **●●●○** | **●●** | **●●●** | **●** | **●** | **●** | **●** | **●** | **●** | **●** | **19**  **19**  **20**  **18**  **18**  **20**  **20**  **20**  **16**  **20**  **20**  **20**  **20**  **19**  **19**  **19**  **20**  **19**  **19**  **20**  **18**  **20**  **19**  **20**  **18**  **18**  **20**  **20**  **20**  **16**  **20**  **20**  **20**  **20**  **19**  **19**  **19**  **20**  **19**  **19**  **20**  **18**  **20**  **19** |
| Hou | **●** | **●●** | **●** | | **●●●○** | **●●** | **●●●** | **●** | **●** | **●** | **●** | **●** | **●** | **●** | **19** |
| Kwak | **●** | **●●** | **●** | | **●●●●** | **●●** | **●●●** | **●** | **●** | **●** | **●** | **●** | **●** | **●** | **20** |
| Li | **●** | **●●** | **●** | | **●●●○** | **●●** | **●●●** | **●** | **○** | **●** | **●** | **●** | **●** | **●** | **18** |
| Luo | **●** | **●●** | **●** | | **●●●○** | **●●** | **●●●** | **●** | **○** | **●** | **●** | **●** | **●** | **●** | **18** |
| Luo | **●** | **●●** | **●** | | **●●●●** | **●●** | **●●●** | **●** | **●** | **●** | **●** | **●** | **●** | **●** | **20** |
| Mi | **●** | **●●** | **●** | | **●●●●** | **●●** | **●●●** | **●** | **●** | **●** | **●** | **●** | **●** | **●** | **20** |
| Rong | **●** | **●●** | **●** | | **●●●●** | **●●** | **●●●** | **●** | **●** | **●** | **●** | **●** | **●** | **●** | **20** |
| Skidmore | **●** | **●●** | **●** | | **●●○○** | **●●** | **●●●** | **●** | **○** | **●** | **●** | **●** | **○** | **●** | **16** |
| Sun | **●** | **●●** | **●** | | **●●●●** | **●●** | **●●●** | **●** | **●** | **●** | **●** | **●** | **●** | **●** | **20** |
| Tang | **●** | **●●** | **●** | | **●●●●** | **●●** | **●●●** | **●** | **●** | **●** | **●** | **●** | **●** | **●** | **20** |
| Wang | **●** | **●●** | **●** | | **●●●●** | **●●** | **●●●** | **●** | **●** | **●** | **●** | **●** | **●** | **●** | **20** |
| Wang | **●** | **●●** | **●** | | **●●●●** | **●●** | **●●●** | **●** | **●** | **●** | **●** | **●** | **●** | **●** | **20** |
| Wang | **●** | **●●** | **●** | | **●●●○** | **●●** | **●●●** | **●** | **●** | **●** | **●** | **●** | **●** | **●** | **19** |
| Wen | **●** | **●●** | **●** | | **●●●○** | **●●** | **●●●** | **●** | **●** | **●** | **●** | **●** | **●** | **●** | **19** |
| Xiang | **●** | **●●** | **●** | | **●●●●** | **●●** | **●●○** | **●** | **●** | **●** | **●** | **●** | **●** | **●** | **19** |
| Xu | **●** | **●●** | **●** | | **●●●●** | **●●** | **●●●** | **●** | **●** | **●** | **●** | **●** | **●** | **●** | **20** |
| Yao | **●** | **●●** | **●** | | **●●●●** | **●●** | **●●○** | **●** | **●** | **●** | **●** | **●** | **●** | **●** | **19** |
| Yue | **●** | **●●** | **●** | | **●●●●** | **●●** | **●●●** | **●** | **○** | **●** | **●** | **●** | **●** | **●** | **19** |
| Zhang | **●** | **●●** | **●** | | **●●●●** | **●●** | **●●●** | **●** | **●** | **●** | **●** | **●** | **●** | **●** | **20** |
| Zhang | **●** | **●●** | **●** | | **●●○○** | **●●** | **●●●** | **●** | **●** | **●** | **●** | **●** | **●** | **●** | **18** |
| Zhang | **●** | **●●** | **●** | | **●●●●** | **●●** | **●●●** | **●** | **●** | **●** | **●** | **●** | **●** | **●** | **20** |
| Zhang | **●** | **●●** | **●** | | **●●●○** | **●●** | **●●●** | **●** | **●** | **●** | **●** | **●** | **●** | **●** | **19** |

**Table S3. Objective assessment of quality of included studies. (Group CBF)**

|  | **Sample characteristics (10)** | | | | | **Methodology and reporting (10)** | | | | | | | | |  |
| --- | --- | --- | --- | --- | --- | --- | --- | --- | --- | --- | --- | --- | --- | --- | --- |
| **Study** | standardised diagnostic criteria | Important demographic data (age and gender) | Healthy comparison subjects | Important clinical variables | Sample size per group > 10 | | Whole brain analysis was automated | Magnet strength at least 1.5T | At least 5 minutes of resting state acquisition | Whole brain coverage of resting scans | The acquisition and preprocessing techniques were clearly described | Coordinates reported in a standard space | FDR, FWE or permutation-based methods | Conclusions were consistent with the results | Quality score |
| Arslan | **●** | **●●** | **●** | **●●●●** | **●●** | **●●●** | | **●** | **○** | **●** | **○** | **●** | **●** | **●** | 18 |
| Barzgari | **●** | **●●** | **●** | **●●●○** | **●●** | **●●●** | | **●** | **○** | **●** | **●** | **●** | **●** | **●** | 18 |
| Jia | **●** | **●●** | **●** | **●●●○** | **●●** | **●●●** | | **●** | **●** | **●** | **●** | **●** | **●** | **●** | 19 |
| Lin | **●** | **●●** | **●** | **●●●●** | **●●** | **●●●** | | **●** | **○** | **●** | **●** | **●** | **●** | **●** | 18 |
| Lin | **●** | **●●** | **●** | **●●●●** | **●●** | **●●●** | | **●** | **○** | **●** | **●** | **●** | **●** | **●** | 19 |
| Shang | **●** | **●●** | **●** | **●●●●** | **●●** | **●●●** | | **●** | **○** | **●** | **●** | **●** | **●** | **●** | 19 |
| Suo | **●** | **●●** | **●** | **●●●●** | **●●** | **●●●** | | **●** | **●** | **●** | **●** | **●** | **●** | **●** | 20 |
| Zhao | **●** | **●●** | **●** | **●●●●** | **●●** | **●●●** | | **●** | **●** | **●** | **●** | **●** | **○** | **●** | 19 |

| **Table S4. The Joanna Briggs Institute (JBI) Critical Appraisal Checklist for analytical cross-sectional study (last amended in 2017)**  **Website:** https://joannabriggs.org/critical_appraisal_tools  https://wiki.joannabriggs.org/display/MANUAL/Appendix+7.5+Critical+appraisal+checklist+for+analytical+cross-sectional+studies | | | | |
| --- | --- | --- | --- | --- |
| Major Components | Response options | | | |
| 1. Were the criteria for inclusion in the sample clearly defined? | Yes | No | Unclear | Not applicable |
| 2. Were the study subjects and the setting described in detail? | Yes | No | Unclear | Not applicable |
| 3. Was the exposure measured in a valid and reliable way? | Yes | No | Unclear | Not applicable |
| 4. Were objective, standard criteria used for measurement of the condition? | Yes | No | Unclear | Not applicable |
| 5. Were confounding factors identified? | Yes | No | Unclear | Not applicable |
| 6. Were strategies to deal with confounding factors stated? | Yes | No | Unclear | Not applicable |
| 7. Were the outcomes measured in a valid and reliable way? | Yes | No | Unclear | Not applicable |
| 8. Was appropriate statistical analysis used? | Yes | No | Unclear | Not applicable |
| Overall appraisal: Include □ Exclude □ Seek further info □ | | | | |

| **Table S5. Results of JBI scoring** | | | | |
| --- | --- | --- | --- | --- |
| **Analysis of ALFF** | |  | **Analysis of CBF** | |
| **Study** | **JBI score** |  | **Study** | **JBI score** |
| Harrington et al. | 15 |  | Arslan et al. | 14 |
| Hou et al. | 15 |  | Barzgari et al. | 13 |
| Kwak et al. | 16 |  | Jia et al. | 15 |
| Li et al. | 13 |  | Lin et al. | 14 |
| Luo et al. | 14 |  | Lin et al. | 15 |
| Luo et al. | 16 |  | Shang et al. | 14 |
| Mi et al. | 16 |  | Suo et al. | 16 |
| Rong et al. | 16 |  | Zhao et al. | 15 |
| Skidmore et al. | 12 |  |  |  |
| Sun et al. | 16 |  |  |  |
| Tang et al. | 16 |  |  |  |
| Wang et al. | 16 |  |  |  |
| Wang et al. | 16 |  |  |  |
| Wang et al. | 15 |  |  |  |
| Wen et al. | 15 |  |  |  |
| Xiang et al. | 15 |  |  |  |
| Xu et al. | 16 |  |  |  |
| Yao et al. | 14 |  |  |  |
| Yue et al. | 15 |  |  |  |
| Zhang et al. | 15 |  |  |  |
| Zhang et al. | 14 |  |  |  |
| Zhang et al. | 16 |  |  |  |
| Zhang et al. | 14 |  |  |  |

**Table S6. Radiographic characteristics of the studies included in the meta-analysis. (Group ALFF)**

| **Study** | **Scanner** | **Scan duration** | **Software** | **Indicator** | **Frequency range (Hz)** | **Smoothing kernel (mm)** | **Threshold (method)** | **GMV correction** | **Quality score^a^** |
| --- | --- | --- | --- | --- | --- | --- | --- | --- | --- |
| Harrington | 3T | 6min | SPM, AFNI,3dRSFC | ALFF | 0.01-0.1 | 4 | p＜0.05 (FDR corrected) | NO | 19 |
| Hou | 3T | 8min | SPM8, REST | ALFF | 0.01-0.73 | 3 | p＜0.05 (FWE corrected) | NO | 19 |
| Kwak | 3T | 8min | SPM5, | ALFF | 0.01-0.08 | 8 | p＜0.05 (Bonferroni corrected) | NO | 20 |
| Li | 3T | / | SPM, DARASF, REST | ALFF | 0.01-0.08 | 6 | p＜0.05 (AlphaSim corrected) | YES | 18 |
| Luo | 1.5T | / | SPM, DPABI, REST | ALFF | 0.01-0.1 | 4 | p＜0.05 (FWE corrected) | YES | 18 |
| Luo | 3T | 6min40s | SPM8, REST | ALFF | 0.01-0.08 | 8 | p＜0.05 (FWE corrected) | NO | 20 |
| Mi | 3T | 8min | SPM12, DPABI, REST | ALFF | 0.01-0.08 | 6 | p＜0.05 (AlphaSim corrected) | YES | 20 |
| Rong | 3T | 6min | SPM12, REST | ALFF | 0.01-0.08 | 4 | p＜0.05 (GRF corrected) | YES | 20 |
| Skidmore | 3T | / | AFNI | ALFF | 0.01-0.08 | 6 | p＜0.005 uncorrected | NO | 16 |
| Sun | 3T | ＞5min | SPM12, DPABI | ALFF | 0.01-0.1 | 4 | p＜0.05 (FWE corrected) | YES | 20 |
| Tang | 3T | 6min | DAPRSF, REST | ALFF | 0.01-0.08 | 8 | p＜0.01 (Multiple Comparison corrected) | NO | 20 |
| Wang | 3T | ＞5min | SPM8, DPABI | ALFF | 0.01-0.08 | 6 | p＜0.05 (AlphaSim corrected) | YES | 20 |
| Wang | 3T | ＞5min | SPM8, DAPRSF | ALFF | 0.01-0.08 | 4 | p＜0.01 (AlphaSim corrected) | YES | 20 |
| Wang | 3T | ＞5min | SPM12, DPARSF | ALFF | 0.01-0.1 | 4 | p＜0.05 (FWE corrected) | YES | 19 |
| Wang | 3T | ＞5min | SPM12, DPARSF | ALFF | 0.01-0.1 | 4 | p＜0.05 (FWE corrected) | YES | 19 |
| Wen | 3T | 7min | SPM8, REST | ALFF | 0.01-0.08 | 5 | p＜0.05 (Multiple Comparison corrected) | NO | 19 |
| Xiang | 3T | 6min58s | SPM8 | ALFF | 0.01-0.08 | 6 | p＜0.05 (AlphaSim corrected) | NO | 19 |
| Xu | 3T | 2min50s | SPM12, REST | ALFF | 0.01-0.08 | 6 | p＜0.05 (GRF corrected) | YES | 20 |
| Yao | 3T | 6min36s | SPM8, REST | ALFF | 0.01-0.08 | 4 | p＜0.05 (Multiple Comparison corrected) | NO | 19 |
| Yue | 3T | / | SPM, REST | ALFF | 0.01-0.08 | 6 | p＜0.01 (AlphaSim corrected) | NO | 19 |
| Yue | 3T | / | SPM, REST | ALFF | 0.01-0.08 | 6 | p＜0.01 (AlphaSim corrected) | NO | 19 |
| Zhang | 3T | 6min10s | SPM, DPABI | ALFF | 0.01-0.08 | 6 | p＜0.05 (GRF corrected) | YES | 20 |
| Zhang | 3T | 8min | SPM8, REST | ALFF | 0.01-0.08 | 8 | p＜0.05 (FWE corrected) | NO | 18 |
| Zhang | 3T | 8min | SPM8, DPARSF | ALFF | 0.01-0.08 | 6 | p＜0.05 (AlphaSim corrected) | NO | 20 |
| Zhang | 3T | 6min | SPM8, DPARSF | ALFF | 0.01-0.08 | 4 | p＜0.01 (AlphaSim corrected) | YES | 19 |

GMV, gray matter volume; 3dRSFC, 3d resting-state functional connectivity; SPM, statistical parametric mapping; DPARSF, data processing assistant for resting-state fMRI; REST, the resting-state fMRI data analysis toolkit; DPABI, data processing and analysis for brain imaging; AFNI, analysis of functional neuroimages; FDR, false discovery rate; GRF, Gaussian random field; FWE, family-wise error.

^a^ Maximum score of 20 for each study.

**Table S7. Radiographic characteristics of the studies included in the meta-analysis. (Group CBF)**

| **Study** | **Scanner** | **Indicator** | **Scan**  **duration** | **Software** | **Smoothing**  **kernel(mm)** | **Labeling**  **Duration (ms)** | **Post label**  **Delay (ms)** | **Threshold (method)** | **GMV correction** | **Quality score** |
| --- | --- | --- | --- | --- | --- | --- | --- | --- | --- | --- |
| Arslan | 3T | CBF | 4min8s | SPM12, FSL | / | / | / | p＜0.05 (FWE corrected) | NO | 18 |
| Arslan | 3T | CBF | 4min8s | SPM12, FSL | / | / | / | p＜0.05 (FWE corrected) | NO | 18 |
| Barzgari | 3T | CBF | 4min44s | SPM12 | 8 | / | 2025 | p＜0.05 (AlphaSim corrected) | NO | 18 |
| Jia | 3T | CBF | 8min | SPM12 | 6 | 700 | 1100 | p＜0.05 (FWE corrected) | YES | 19 |
| Jia | 3T | CBF | 8min | SPM12 | 6 | 700 | 1100 | p＜0.05 (FWE corrected) | YES | 19 |
| Lin | 1.5T | CBF | 4min | SPM8 | 6 | / | 1525 | p＜0.001 uncorrected | NO | 18 |
| Lin | 1.5T | CBF | 4min | SPM8 | 6 | / | 1525 | p＜0.05 (AlphaSim corrected) | NO | 19 |
| Lin | 1.5T | CBF | 4min | SPM8 | 6 | / | 1525 | p＜0.05 (AlphaSim corrected) | NO | 19 |
| Shang | 3T | CBF | 4min | SPM12 | 8 | 1500 | 2025 | p＜0.05 (FWE corrected) | YES | 19 |
| Suo | 3T | CBF | 5min54s | SPM8 | 6 | 1500 | 1800 | p＜0.05 (AlphaSim corrected) | NO | 20 |
| Suo | 3T | CBF | 5min54s | SPM8 | 6 | 1500 | 1800 | p＜0.05 (AlphaSim corrected) | NO | 20 |
| Zhao | 1.5T | CBF | 5min24s | SPM8 | 8 | 1600 | 1525 | p＜0.001 uncorrected | NO | 19 |

GMV, gray matter volume; SPM, statistical parametric mapping; FSL, functional MRI of the brain software library; FWE, family-wise error.

^a^ Maximum score of 20 for each study.
